# *Mentoring up* for early career investigators: Empowering mentees to proactively engage in their mentoring relationships

**DOI:** 10.1017/cts.2024.524

**Published:** 2024-04-22

**Authors:** Fátima Sancheznieto, Pamela Asquith, Adriana Baez Bermejo, Emma A. Meagher, Christine E. Pfund

**Affiliations:** 1 Institute for Clinical and Translational Research, University of Wisconsin-Madison, Madison, WI, USA; 2 Departments of Pharmacology and Otolaryngology, University of Puerto Rico School of Medicine, San Juan, Puerto Rico; 3 Institute for Translational Medicine and Therapeutics, Perelman School of Medicine, Philadelphia, PA, USA

**Keywords:** Curricular development, mentorship education, mentee training, mentoring up, professional development

## Abstract

**Introduction:**

Effective mentorship is recognized as critical for the professional development of clinical and translational investigators. Evidence-based mentorship training prompted the development of training for mentees at early career stages who are navigating both mentor and mentee roles. The curriculum titled, *Mentoring Up* for Early Career Investigators, recognizes the importance of building mentee self-efficacy across proactive mentorship skills and competencies.

**Methods:**

*Mentoring Up for Early Career Investigators* curriculum is based on the research mentor training approach in Entering Mentoring. Pilot implementations of *Mentoring Up* at the University of Wisconsin-Madison and University of Pennsylvania had positive training outcomes for KL2 Scholars. Subsequently, Mentoring Up was implemented and evaluated at several other institutions. For 26 implementations longer than 4 hours, data were collected on trainee demographics, satisfaction with training, skill gains across mentorship competencies, and the intent to change mentoring behaviors following training.

**Results:**

88% of participants rated the mentee training as valuable. Significant skill gains were reported across all mentorship competencies following training. 77% reported specific plans to change or augment their mentoring behaviors because of the training. The majority aligned with mentorship skill competencies (*aligning expectations, effective communications*) or mentoring up strategies (*voicing needs, setting boundaries, communicating proactively*).

**Conclusion:**

*Mentoring Up* training is effective in advancing mentee skills and promoting strategies to be more proactive in getting their mentoring needs met. *Mentoring Up* offers an expansion to the suite of mentorship education and resources to support the career advancement of all in the translational science workforce.

## Introduction

Effective mentorship is recognized as critical to the professional development of those engaged in biomedical research careers, including clinical and translational investigators [1–4]. Mentees with strong mentorship experiences have an enhanced science identity, sense of belonging, self-efficacy, career satisfaction, and research productivity [5–7]. On the other hand, mentees who experience negative mentorship report lower job satisfaction, increased stress, and poor outcomes [4,8,9]. Given the importance of mentorship to career success [10–12], mentorship education is key to train both mentees and mentors in evidence-based mentorship skills. The benefits for mentors who complete formal research mentor training have been well documented [13–16].

Similarly, there has been growing recognition that mentees can be empowered to better navigate challenging professional and personal domains and acquire skills that optimize their mentoring relationships [17,18]. In other words, mentees can be given tools to proactively shape their mentoring relationships into working alliances that benefit both mentor and mentee [4,18]. Under this premise, mentoring relationships are understood not as unidirectional, but rather collaborative processes in which mentees and mentors take part in reciprocal activities such as planning, reflecting, questioning, and problem-solving [18]. An extensive review of the mentorship literature conducted by the National Academies of Science, Engineering, and Mathematics aligns with this view, defining mentorship as “a professional, working alliance in which individuals work together over time to support the personal and professional growth, development, and success of the relational partners through the provision of career and psychosocial support” [4].

Defining mentorship as a working alliance elevates the relational nature of mentorship and affirms the importance of training mentees as well as mentors. As an example, *Entering Research* [19] was developed to teach research and mentorship skills to undergraduates and graduates starting their research careers. Students in the *Entering Research* course reported statistically significant gains in their skills, knowledge, and confidence as researchers compared with a control group of students, who also were engaged in undergraduate research but not enrolled in this course [20]. This evidence, alongside growing interest from program directors of NIH Mentored Training Programs (e.g., KL2, K12, T32, and TL1) to integrate mentorship education into their portfolios, prompted the development of training similar to *Entering Research* and *Entering Mentoring* but tailored to postdoctoral fellows (postdocs) and early career clinicians and faculty. Because scientists at these career stages actively navigate transitions from early careers to greater independence which often includes them playing roles as both mentees and mentors, they require specific considerations for mentor-mentee training.

The concept of “mentoring up” was adapted from the business concept of “managing up” [21] and has been described as “a concept that empowers mentees to be active participants in their mentoring relationships by shifting the primary emphasis from the mentors’ responsibilities in the mentor-mentee relationship to acknowledging the mentees’ contributions of equivalent importance” [18]. Aligning well with the unique positionality and training needs of early career scientists, this concept of “mentoring up” was applied to the development of a mentee training program for early career scientists and faculty. The purpose of this paper is to describe the development, testing, and implementation of a curriculum, *Mentoring Up for Early Career Investigators* [22], focused primarily on mentees who are early career investigators.

## Materials and methods

The structure and format of the *Mentoring Up for Early Career Investigators* curriculum is based on the research mentor training approach in *Entering Mentoring* that has been well documented as an evidence-based curriculum [14]. The EM curriculum defines a core set of mentoring competencies and learning objectives for each competency that can be applied by mentees to navigate a variety of mentoring situations and challenges. The training utilizes a process-based approach [23] that employs case studies and group discussions to promote collective learning. Mentoring tools and resources are also shared with participants.

The foundational mentoring competencies in the *Mentoring Up* curriculum are complementary to those defined in the original *Entering Mentoring* curricula with the addition of two modules “Enhancing Work-Life Integration” [24] and “Building Research Self-Efficacy” [25]. Each of the eight mentee competencies (“Maintaining Effective Communication,” “Aligning Expectations,” “Enhancing Work-Life Integration,” “Addressing Equity and Inclusion,” “Building Research Self-Efficacy,” “Achieving Independence,” and “Seeking Professional Development”) is accompanied by a set of specific mentoring up strategies that mentees may employ to be proactive in their mentoring relationships. These strategies for each competency are outlined in Supplementary Table S1.

As with other *Entering Mentoring* curricula, the *Mentoring Up* curriculum is designed as a facilitator guide with participant activities and resources aligned with each learning objective. Participant materials and detailed facilitator strategies are included in the curriculum. Training activities were customized to support mentee skill development to effectively “mentor up” in their mentorship relationships. Case studies were written or adapted to focus on mentoring scenarios or dilemmas mentees might encounter (e.g., “Balancing Multiple Mentors’ Expectations”). Tools to support participants' development as proactive mentees were also included. For example, participants engage with self-assessment tools to help them discern and prioritize their mentoring needs. With that information, mentees are introduced to the value of mentoring networks. Mentoring networks are constellations of mentors, mentoring relationships, and mentorship resources that a mentee can engage for support [4,26,27]. Mentees learn about the concept of mentoring maps [28] and work on the design of their own mentoring networks as depicted in Supplementary Figure S1.

### Pilot implementations

Once the *Mentoring Up* curriculum was complete and authors agreed it was ready for piloting, the training was first implemented by CP in 2015 at the UW-Madison Institute for Clinical and Translational Research (ICTR) for a cohort of assistant professors in their first year in a KL2 Program (NIH KL2 awards support mentored research career development for early career faculty who are conducting basic, translational and/or clinical research). The training was then implemented by EM at the University of Pennsylvania’s Institute for Translational Medicine and Therapeutics for late-stage postdocs and early career faculty engaged in a similar program in 2016. Post-training surveys were administered to both the participants and the facilitators via Qualtrics by the UW ICTR mentorship evaluation team. Evaluation reports were reviewed and discussed by the curriculum developers. Survey feedback from both participants and facilitators prompted a few minor adaptations to curricular activities and clearer facilitator guidelines to enhance the training. The curriculum then became available for wider implementation.

### Implementation

**Figure 1.**
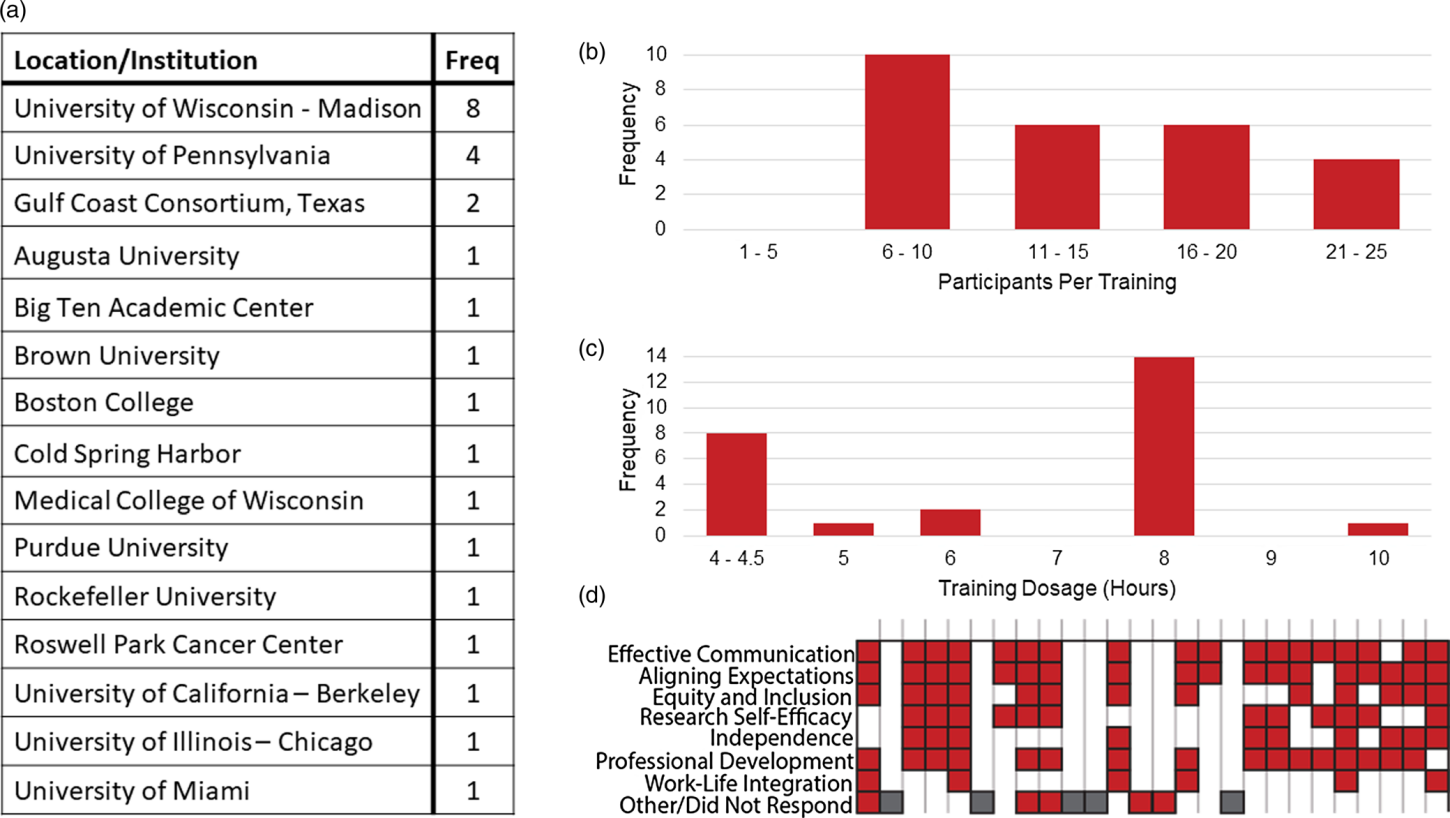
Description of workshops. a. Geographical location or institution at which workshops were held, with frequencies for the number of workshops held at each site. b. Distribution of participants that attended the training, with participant numbers on the × axis and frequency of the workshops with that many participants on the *y* axis. A total of 354 participants attended the workshops. c. Training dosage for workshop in hours, with workshop hours on the × axis and frequency of workshops on the *y* axis. Note: Workshops less than 4 hours long were excluded from our analysis. d. Distribution of modules taught at each workshop. Each column across the *X* axis represents an individual workshop. Red boxes represent the modules covered in a workshop. Gray boxes represent 5 workshops for which the mentorship competencies covered are unknown. Workshops varied in the modules that were implemented given time restrictions and/or specific site needs.

Sixty-two implementations of the *Mentoring Up for Early Career Investigators* were delivered between September 2015 and August 2021 for which we collected evaluation data. Of these, we excluded workshops that were less than 4 hours (*n* = 34) in length or those that were implemented with mentors and mentees as a combined mentor-mentee training (*n* = 2). Of the resulting twenty-six implementations, nineteen were held in person and 7 were conducted synchronously online due to the COVID-19 pandemic. The 26 implementations took place at 14 institutions in addition to the BigTen Center of the Midwest (Fig. 1a). Of the 14 institutions, 7 are Clinical and Translational Science Award (CTSA) Hubs (UW-Madison, University of Pennsylvania, Medical College of Wisconsin, Purdue, Rockefeller, UC-Berkley and University of Illinois-Chicago).

Most of the *Mentoring Up* workshops included six to ten participants while some enrolled as many as twenty-five (Fig. 1b). The length of training sessions was typically four or eight hours (Fig. 1c), within the range of dosages of training found to be most effective [29]. Training dosage variations across implementations reflect whether the site chose to offer the complete curriculum or a subset of the mentoring competencies (e.g., “Maintaining Effective Communication,” *“*Aligning Expectations,*”* “Addressing Equity and Inclusion”). There were six implementations that also included the “Work-Life Integration” module (Fig. 1d).

### Survey dissemination and data collection

A total of 354 participants completed the training during the time period reported, and of 226 participants who responded to some or all of the post-implementation survey items, 183 consented to have their survey data used for research. Ethical approval was granted by the University of Wisconsin Institutional Review Board, protocol numbers 2016-0458, 2017-0026, 2017-1336. Surveys were originally sent to participants through Qualtrics and then switched to an in-house survey-building and data collection platform. Surveys were sent immediately following training and remained open for a month before closing for data cleaning and analysis. Survey response rates varied across the 26 implementations, with an average of 67.6% ± 23.6% response rate. In addition to demographic questions, workshop evaluation questions (e.g., *facilitator effectiveness, overall training satisfaction, recommend training to a colleague, good use of time*) the survey included the validated Mentorship Competency Assessment (MCA) items [30] to survey participants’ self-assessment of their mentoring up skills before and after the training. The MCA uses 26 items to assess skills across six mentoring competencies (Communication, Understanding, Professional Development, Expectations, Independence, and Diversity) and therefore allows us to test improvements in mentoring skills specifically addressed in the curriculum. Because the MCA was originally developed for mentors to reflect on their mentoring skills or for mentee’s to reflect on their mentor’s skills, we adapted the MCA prompt and items so that participants could reflect on their own skills as mentees. The prompt was changed as follows: *Please rate how skilled you feel you were BEFORE attending the research mentee training, and how skilled you feel you are NOW in each of the following areas: (Think about your skills with your primary mentor).* Items were adapted to reflect mentee skills accordingly. For example, where the original MCA reads *Providing constructive feedback* for one item, the adapted version used in this study reads *Receiving constructive feedback*. This mentee version of the MCA scale is currently undergoing validation. To assess participants’ skill increases related to self-efficacy, we employed the same scale used when assessing the original “Building Research Self-Efficacy” Module [25]. Similar to the MCA, the items were adapted for mentees in the Mentoring Up training. De-identified data were stored in a secure server, and SPSS software was used to clean and aggregate the data into one large dataset.

### Quantitative analysis

Graphs were created and statistics (arithmetic mean, standard deviation) were calculated using Microsoft Excel. One graph, a box plot, was created using the R data visualization package, ggplot2 (https://ggplot2.tidyverse.org/index.html), and Adobe Illustrator was used to illustrate the modules implemented during each training. Population means of Mentoring Competency Assessment (MCA) scores both before and after training from participants across three career stages (Assistant Professor, Postdoctoral Fellow, Graduate Student) were compared using the Kruskal-Wallis test for comparison of multiple population means from non-parametric datasets. There were no statistical differences in MCA means across career stages so MCA scores from all career stages were used for further analysis as a single population. MCA scores derived from participant self-assessment of a mentoring competency before and after completing the training were compared using the Wilcoxon Rank Sum Test for paired samples in a non-parametric dataset. *** denote a p-value below 0.001. Statistical analyses were run using SPSS version 28.

### Qualitative analysis

Short-answer responses to survey questions were analyzed using a Grounded Theory approach [31]. The goal of utilizing this approach was to gain deeper insight into participant experiences with the training rather than develop a new theoretical framework. A similar approach to simple, short-answer response coding has been used previously by the authors [32,33]. For the first round of iterative coding, FS employed open coding followed by code consolidation [34]. Using this codebook, FS and PA independently coded and then met to discuss and code to consensus, allowing for conversations regarding the data and to prevent one author’s narrative or interpretation of the data and codes to override that of the other.

## Results

### Participant career stage and demographics

Given the targeted implementation to train early career stage investigators, the majority of participants reported being at either the Assistant Professor, Graduate Student, or Postdoc stage of their careers (Table 1a). Survey respondents reporting race, ethnicity, and gender identified as white (*n* = 86) followed by Asian (*n* = 39) and Hispanic/Latinx (*n* = 18; Table 1b) and as women (*n* = 82; Table 1c).

**Table 1.** Participant information

	Frequency
**a. Career Stage/Title**	
Assistant Professor	45
Graduate Student	43
Postdoc	35
Scientists/Researcher	14
Medical Student	9
Clinical Fellow	7
Lecturer/Instructor	4
Clinical Instructor	3
Assistant Dean	2
Other	9
**b. Race/Ethnicity**	
White	86
Asian	39
Hispanic/Latinx	18
Black/African American	11
American Indian/Alaskan Native	1
Native Hawaiian/Pacific Islander	0
Other	8
**c. Gender**	
Women	82
Men	67
Nonbinary	1
Prefer Not to Report	1

1a. Survey respondent career stage, listed in order of most to least common (*n* = 171). 1b. Respondent race and ethnicity (*n* = 163). 1c. Respondent gender (*n* = 151). Please note that for all of these questions, participants could choose more than one option.

### Training effectiveness and satisfaction

Participants reported high satisfaction with training, with 89.6% survey respondents reporting that their facilitators were either “effective” or “very effective” on a 5-point Likert-type scale. 87.9% of survey respondents claimed that the training was a valuable use of their time while 80.9% would recommend the training to a colleague (Table 2).

**Table 2.** Participant satisfaction with *mentoring up* training

	Percentage
Facilitator Effectiveness	89.6% (Effective or Very Effective)
Valuable Use of Time	87.9% (Yes)
Recommend to a Colleague	80.9% (Yes)
Planned Behavior Changes	77% (Yes)

Percentage of respondents who: a. rated the workshops as effective or very effective; b. reported that the workshop was a valuable use of their time; c. would recommend the workshop to a colleague; d. and had either made changes or planned to make changes in their mentoring relationships.

### Skill gains and planned behavior changes

**Figure 2.**
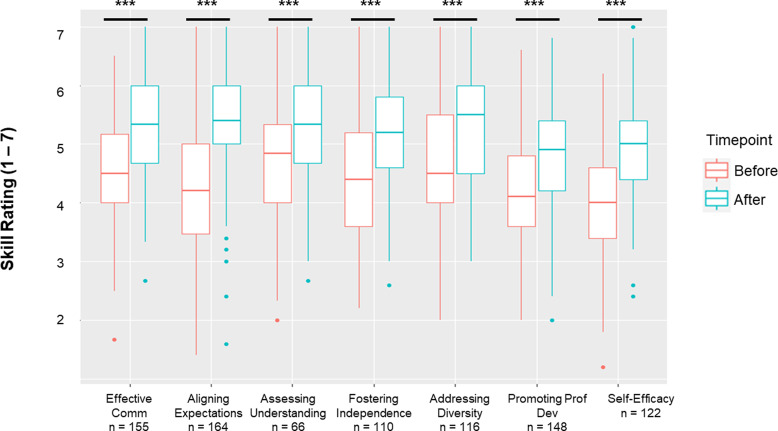
Mentor competency assessment (MCA) and building self-efficacy skills. Immediately following workshop participation, participants were asked to rate their skill level across various mentorship competencies, including competencies related to building self-efficacy. They were prompted to consider their skill both BEFORE taking the training and AFTER taking the training. Boxplots for the distribution of competency mean scale scores are provided. Wilcoxon rank sum test for paired non-parametric samples showed significant changes in the reported competency levels before and after training. Sample size is reported below each competency. *p* < 0.001 for all competency subscale comparisons.

Participants were surveyed to assess their skill level retrospectively for each of the mentoring competencies prior to completing the *Mentoring Up* training and afterward. Significant skill gains were reported by participants across all of the mentoring competencies as a result of completing the training (Fig. 2).

Participants were asked to describe any changes they had made or planned to make in their mentoring relationships as a result of the training. Of participants surveyed, 77% reported behavior changes. The types of changes shared most commonly reflected the learning objectives and strategies learned for each competency in the *Mentoring Up* curricula, such as *Maintaining Effective Communication* and *Aligning Expectations* (Table 3). Below are a few examples of changes that mentees reported they plan to make:

**Table 3.** Planned changes due to workshop

Code	Frequency (Total *n* = 124)	Example
Effective Communication	29	*I plan to incorporate active listening into my mentoring relationships better than I have been doing*
“Mentoring Up”/Proactive (voicing needs/setting boundaries, taking initiative, etc.)	26	*I have begun “mentoring up” up (sic) a much greater degree and have tried to be better about communicating my needs*
Aligning Expectations	25	*Ask clear expectations to make sure we are on the same page*
Intentional Meeting Management	21	*I plan to implement more structure in my meetings with mentors…*
Mentoring Resources and Tools	15	*I plan to use the mentoring guide that was provided us during the session*
Career/Professional Development (goal setting, planning, timelines, etc.)	10	*Been more clear about my career goals*
Expand/Assess Mentor Network	7	*I have began to increase the number of mentors I have*
Fostering Self-Efficacy	5	*I ask myself why I am reluctant to do certain tasks, then try to come up with a strategy to help (e.g., I’ve done it before, seen someone do it before, etc.)*
Soliciting Feedback	2	*I plan to solicit more direct and specific feedback about my performance…*
Work-Life Integration	2	*I’m more likely to implement other experiments for work-life integration after this course*
Delegating	2	*…delegating more to others*
Cultural/Diversity Awareness	2	*More likely to reflect on biases…*
Other	10	
Vague Response	6	
No Planned Changes	5	

Summary and frequency of codes used in qualitative analysis of short-answer responses to the question “Please describe any changes you plan to make as a result of this workshop (*n* = 124). Codes that only corresponded to one answer were grouped together as “Other.” Note that some responses were double-coded to more fully capture the extent of the response. Frequencies will therefore not sum to 124.

*I plan to incorporate active listening into my mentoring relationships better than I have been doing.*

*I’m using the areas from the self-assessment to figure out what my priorities for mentorship are and what needs I need to get met that are not currently.*

*Improve clarity of expectations between myself and my mentors and to make more effective use of our meetings.*

Participants also reported planned changes in behavior that aligned broadly with the concept of mentoring up (e.g., setting boundaries, advocating for oneself, communicating needs) and being more intentional in managing meetings with mentors.

*I have begun “mentoring up” up (sic) to a much greater degree and have tried to be better about communicating my needs.*

*I plan to implement more structure in my meetings with mentors.*

*I will take a more active role. I never really thought of the mentor-mentee relationship as a two way street.*

In many cases, mentees commented on multiple strategies in their response. The example below encapsulates one such response:

*I have been more proactive in engaging and meeting regularly with my mentor. I have also begun to take charge of my own career and development and have been employing “managing up” techniques to effectively get what I need to help me in this endeavor from my mentor. In the future, I plan to discuss expectations and communication styles at the beginning of a mentor-mentee relationship to make sure that they are aligned.*

### Most and least useful aspects of training

When queried about which aspects of the training participants found most useful, 127 participants responded to this question. The most frequent response was the opportunity to engage with their near-peer colleagues in discussion about mentoring experiences and strategies (*n* = 44). The usefulness of specific competencies (*n* = 27), case studies and activities (*n* = 25), or tools and resources provided (*n* = 19) were also cited. When asked what aspects of the training could be improved, the most frequent response included some variant of the length of training, with comments ranging from the workshop either being too short or too long in duration (23 of 119 responses).

### Dissemination

At the conclusion of the pilot phase of the *Mentoring Up for Early Career Investigator*s curriculum, a dissemination plan was implemented to increase awareness and share news about this new curriculum for training early career mentees. Information about the curriculum was also shared by the Institute for Clinical and Translational Research (https://ictr.wisc.edu), Center for the Improvement of Mentored Experiences in Research (https://cimerproject.org/), via the National Research Mentoring Network (https://nrmnet.net/) of mentees and mentors, and via newsletters for trained Entering Mentoring facilitators across the United States, many of whom are at CTSA hubs. In addition, the curriculum was posted on the Center for the Improvement of Mentored Experiences in Research (CIMER) website (https://cimerproject.org/) to make it available for public download. Since its posting on the CIMER website in February of 2021, it has been downloaded by 352 individuals at over 200 institutions. Based on the high level of interest and positive outcomes of the *Mentoring Up for Early Career Investigators* curriculum, a companion curriculum *Mentoring Up for Postdoc Trainees* [35] was adapted with a specific focus to help train postdocs to advance professionally. The curriculum includes useful activities and resources that are strategically designed to help postdocs enhance their relationships with their faculty mentors and launch them into future careers. This curriculum is also available on the CIMER website and since September 2019 has been downloaded by 645 individuals at over 300 institutions.

### Sustainability

The *Mentoring Up for Early Career Investigators* has become a standard programmatic component of mentorship education at the UW-Madison Institute for Clinical and Translational Research and at the University of Pennsylvania, Institute for Translational Medicine and Therapeutics. Newly appointed assistant professors to the KL2 Scholars Program are required to complete this training as part of their professional development at both institutions. Over the years, early career faculty from similar career development programs have been invited to participate. Continuous implementation of the training is achieved by building the pool of trained facilitators [36]. For example, several faculty alumni of the ICTR and Penn KL2 Scholars Program have served as facilitators of the *Mentoring Up* training.

## Discussion

In this paper, we describe the development of a new curriculum, *Mentoring Up for Early Career Investigators* for training early career mentees. Several factors drove the development of this training:

1. Evidence that the Entering Research training was effective for training mentees at the undergraduate level so they could leverage those skills to be more proactive in their mentoring relationships [20]
2. Literature that suggested the concept of “managing up” to improve manager-supervisee relationships could be effectively applied to the mentor-mentee relationship within academia [18]
3. An appreciation of the critical and limited time period for effective mentorship as early career investigators were striving to optimize their participation in the KL2 program to publish their research, build a research team, and apply for independent grant funding [16, 17]

Piloting of the training took place at two institutions over a year-long period (2015–2016) followed by subsequent implementations of the training across several institutions. We report data on 26 mentee trainings (183 participant survey responses total) that were 4 hours or more in length from the years 2015–2021. The results of the quantitative and qualitative data analyses indicated that the *Mentoring Up* curriculum is a valuable training for mentees to develop skills to effectively manage their mentoring relationships. Importantly, there were significant reported skill gains across all mentoring competencies. Skill gains varied depending on competency like what has been described in previous literature on *Entering Mentoring* [14]. Compared to *Entering Mentoring* (aimed at the mentors of undergraduate and graduate students) however, we saw higher increases in population means for various competency skills reported in *Mentoring Up*: communication (+0.75 versus + 0.53 for *Entering Mentoring*), aligning expectations (+1.06 versus + 0.45 for *Entering Mentoring*), and professional development (+0.61 versus + 0.37 for *Entering Mentoring*) [14]. However, given that these curricula were not tested side by side, these results are not entirely comparable. Future studies that directly compare skill gains for mentors in *Entering Mentoring* to those of early career investigators in *Mentoring Up* will more fully ascertain whether scientists in earlier career stages have more to gain from mentor and mentee training than those later in their careers. Directly comparing skill gains at varying career stages would inform cohort approaches to mentorship education such as those used at UW-Madison ICTR: *Mentoring Up* is offered to first- and second-year scholars followed by *Entering Mentoring* (Research Mentor Training) training for third and fourth-year scholars. In theory, this training sequence equips early career faculty to first acquire foundational mentee strategies that they can then build on with completion of mentor training. Whether the curricula complement and build on each other when it comes to skills gains, is the topic of future work.

A majority of participant survey respondents (77%) reported they planned to make changes to their mentoring relationships as a result of completing the training. Unsurprisingly, and mirroring previous data collected from implementations of *Entering Mentoring*, planned behavior changes align closely with the skills and tools provided in the curriculum (aligning expectations, effective communication, etc.) [33]. However, we also observed planned behavior changes that aligned with the mentoring up strategies (being proactive, voicing needs, setting boundaries, etc.). Examples of these planned behavior changes represent a novel set of behavior changes not seen in other related mentor and mentee trainings and demonstrate the value of the curriculum to empower mentees to be more strategic and proactive in their mentoring relationships. This study is limited however, in that the changes in behavior are self-reported and cross-sectional. Without longitudinal sampling of respondents or querying of others who are in mentoring relationships with attendees, there is no way of knowing whether changes in behaviors were perceived by others and how long those changes persisted following training. Ongoing work by our team with the Howard Hughes Medical Institute Gilliam Fellowship [33], for example, queries mentor and mentee skills and relationships at various time points throughout training. Work is ongoing to consider this data in light of mentee career outcomes, and could provide an interesting model by which to test the *Mentoring Up* curricula.

It is important to note that empowering mentees to be proactive in their mentoring relationships should not ignore the uneven power dynamics of most formal academic mentoring relationships that can often result in decreased mentee empowerment, creativity, and initiative [9]. Empowering mentees does not release mentors in positions of power of their responsibility to commit to providing mentees with high-quality mentoring tailored to best meet the needs of the mentee. It also does not release institutions and departments from driving change required to make academic training environments more inclusive and supportive of trainees [37]. As a facilitator, FS grounds her *Mentoring Up for Postdocs* implementations with these caveats and provides extra time and space for mentees to express frustrations with current mentoring relationships and explore strategies and solutions. These practices empower mentees with strategies and alleviate pressures they might feel to either assimilate their academic training environments at the expense of their own identities or to take on the emotional labor of changing those training environments themselves.

The COVID-19 pandemic coincided with a segment of the implementation period reported here (2020–2021), prompting the sudden pivot to remote working environments. In response to this change, developers at UW-Madison CIMER and ICTR adapted the facilitation guide of the *Mentoring Up* curriculum and added a tech support component so that the training could be delivered synchronously online. A recent publication [38] comparing face to face with synchronously online mentor training found that the training mode did not significantly impact participants’ perceived training outcomes. This knowledge has positive implications for wider dissemination of mentorship education afforded by the online option and for more accessible forms of delivering training.

*Mentoring Up for Early Career Investigators* curriculum represents an important contribution to advancing mentorship education and includes an adaptation for postdoc trainees. Attributes of this training program include:

1. Mentee skill development to align and elevate their expectations of mentors
2. Strategies and tools to assess mentoring needs and form mentor networks
3. Building mentee self-efficacy across various skills and competencies

Building skills towards functional and intentional mentorship relationships requires input from both members of the mentor-mentee dyad [4], and the *Mentoring Up* curricula provides valuable additions to the *Entering Mentoring* and *Entering Research* educational portfolio with the intent of providing training that addresses the unique needs of early career scientists. Recently, the MCA was revalidated for mentors taking *Entering Mentoring* [39] and work is ongoing to validate the MCA for early career investigator populations completing *Mentoring Up* training. The expansion of validated tools to measure mentorship skills, alongside continuous improvement to curricula, the development of new evidence-based modules and interventions, and research into the dissemination and implementation of training to improve facilitator capacity, provide promising areas for future work on improving the career and professional development of early career investigators.

With the expansion and diversification of the mentorship education portfolio over the past 15 years (e.g., CIMER, https://cimerproject.org/entering-mentoring/) accompanied by the train-the-trainer initiative to build the capacity of trained facilitators [36] across the United States, opportunities to engage in and offer formal mentorship education have grown significantly. With the evolving evidence base that mentoring skills can be learned by individuals across all experience levels and disciplines, institutions of higher education and federal agencies (e.g., NIH, NSF) are raising the bar on mentor qualifications and are adding mentor training requirements to research and training grant proposals. Experience as a mentor is valuable but often no longer sufficient to qualify as a mentor in federally funded career development programs. Amid this landscape, *Mentoring Up* offers an expansion to the suite of mentorship education and resources to build capacity towards a national culture of intentional, evidence-based mentorship that works for and is inclusive of everyone in the workforce.

## Supporting information

Sancheznieto et al. supplementary material Sancheznieto et al. supplementary material

## Supplementary material

The supplementary material for this article can be found at https://doi.org/10.1017/cts.2024.524.
