# Supplementary material for: Mentoring up for early career investigators: Empowering mentees to proactively engage in their mentoring relationships
Source: J Clin Transl Sci. 2024 Apr 22;8(1):e86. doi: 10.1017/cts.2024.524 (PMC11112430; doi:10.1017/cts.2024.524)
Supplement: Sancheznieto et al. supplementary material [file S2059866124005247sup001.docx]

Mentoring Up for Early Career Investigators Curriculum

Summary of Mentoring Up strategies across competencies

Supplementary Material, Table S1

| **Mentoring Up Strategies** | **Mentoring Competency** |
| --- | --- |
| 1. Determine your mentor’s preferred medium of communication (face-to-face, video-conference, texting, phone, email) and acknowledge if it differs from your own personal preference. 2. Schedule a regular time to meet or check in with your mentor. 3. Keep track and share progress toward project and professional goals, both verbally and in writing. 4. Identify challenges and request your mentor’s advice/intervention when appropriate. 5. Prepare for meetings with your mentor by articulating specifically what you want to get out of the meeting and how you will follow up after the meeting. | **Maintaining Effective Communication** |
| 1. Ask your mentor for their expectations regarding: scholars at your stage of career generally, you as an individual scholar, your research projects. 2. Share your expectations regarding your career as a scholar and professional, and your research projects. 3. Ask your mentor about their primary priorities.    1. Examples: upcoming proposal deadlines, seeking tenure and promotion, teaching, family obligations 4. Share your own priorities with your mentor.    1. Examples: research and writing deadlines, teaching and service responsibilities, family obligations | **Aligning Expectations** |
| 1. Reflect on your life and your own assumptions, needs, and desires. 2. Maintain conversations with those close to you to help you set authentic and realistic priorities. 3. Talk to your mentors about how they manage their work-life integration and their expectations for mentees. 4. Be aware of what you are modeling for others. | **Approaching Work-Life Integration** |
| 1. Seek training and professional development activities to understand the multiple aspects of your own identity and improve your ability to work with diverse communities to build inclusive climates. 2. Be open to seeking out and valuing different perspectives. 3. Pause to evaluate your own assumptions. 4. Engage in honest conversation about individual differences with your mentors and co-workers. 5. Contribute positively to shared understandings and solutions to problems. 6. Talk to peers and mentors when you feel conflicted about the ways in which your personal identity intersects with your academic identity. | **Addressing Equity and Inclusion** |
| 1. Recognize the sources of self-efficacy that work well for you and share them with your mentor. 2. Seek out opportunities in your research experience to build your skills to boost your research self-efficacy. 3. Do you know of others who have done similar research and have been successful? Talk to those researchers and use their actions as a model for your own. Consider your role models and what research skills (and attitudes) are being modeled by them. 4. Be aware of how you cope with research challenges and setbacks; do they make you feel confident about the work that you do? Ask your mentor and others to share strategies for what they do when they hit a wall and how they cope with challenges/setbacks in research. 5. Consider how feedback, both positive and critical, affects your beliefs in your capabilities as a researcher. Ask for specific, constructive feedback from your mentor. | **Building Research Self-Efficacy** |
| 1. Define what it takes to do independent work in your field. 2. Define a series of milestones to independence with your mentor and set goals for meeting these milestones as part of your research plan. 3. Ask peers and mentors to share with you their strategies towards achieving independence. | **Achieving Independence** |
| 1. Create an Individual Development Plan (IDP) to set goals and guide your professional development. 2. Seek out and engage multiple mentors to help you achieve your professional goals. 3. Ask peers and mentors to discuss with you the fears and reservations you may have about pursuing a certain career path. | **Seeking Professional Development** |

Mentoring Up for Early Career Investigators Curriculum

Mapping Your Mentor Network

Supplementary Material, Figure S1

**Mentoring Up: Aligning Expectations**

Mapping Your Mentor Network

Using the example of the mentor map below and the insights gained from specifying roles for your mentors and prioritizing your mentoring needs, develop your own mentor map to aid in identifying and navigating your own mentoring relationships, as well as to establish new ones based on your unmet mentoring needs. Be sure to specifically label which needs will be addressed by each mentor and resource node. Where unknown nodes exist or relationships are yet to be established, take time to discuss within your group effective ways to move forward.


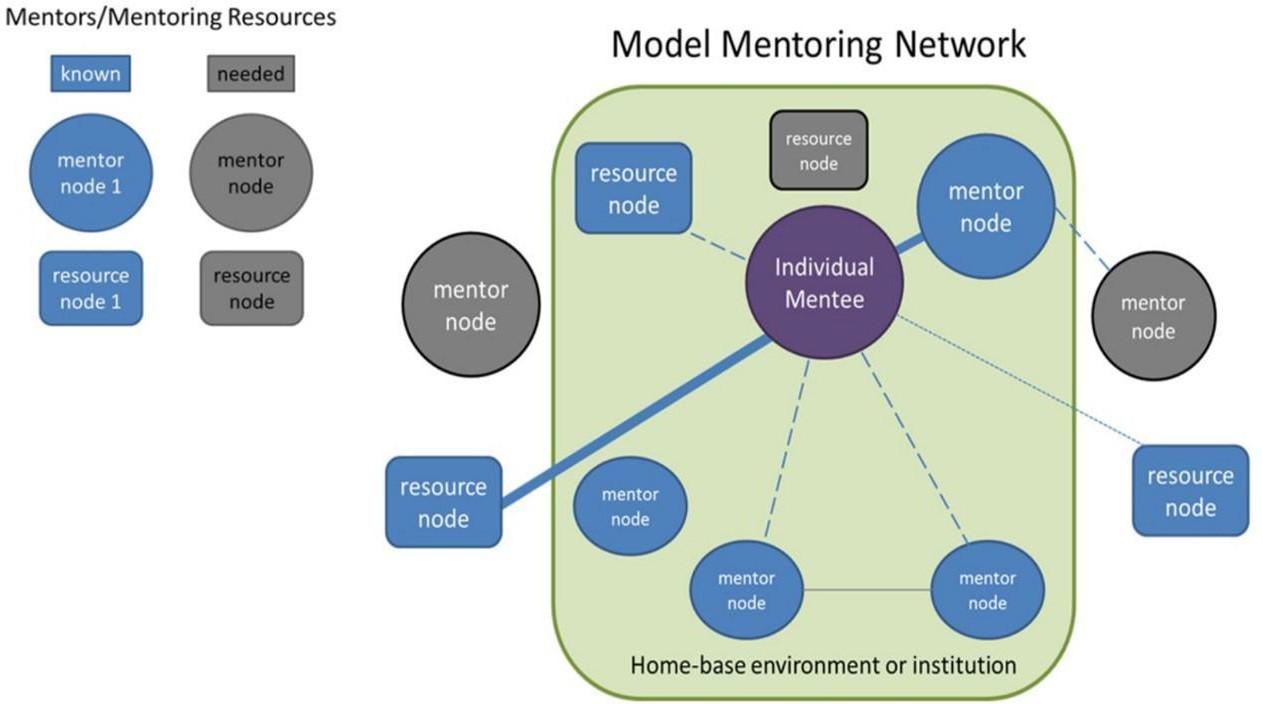


#### Mentoring Network Mapping Model.

Start out by placing a node that represents you in the center of the map. Then, draw a shape around it to represent your local department or institution (Green box in the example map above). Think about which mentors (circles) and mentoring resources (rectangles) are currently available to you, then draw them into your mentor map. Make use of the distance and connections among nodes to represent the relationships and interactions between you and your mentors or your engagement with a particular resource (thinner, dotted lines and thicker solid lines representing the strength of the relationship, and distance representing professional, physical, or emotional distance to a particular mentor). Finally, think about which of your mentoring needs continue to be unmet, and draw some differently colored nodes to represent these (grey nodes in the above example). What are some effective strategies for identifying and connecting with these nodes in your mentor network?

*Resourced from Montgomery, B. 2017 Mapping a Mentoring Roadmap and Developing a Supportive Network for Strategic Career Advancement, SAGE Open.*
